# C-851-05. Enzymatic Debridement Combined with Autologous Micro-grafting and Bioactive Dressings versus SOC for Deep Dermal Burns

**DOI:** 10.1093/jbcr/irag033.057

**Published:** 2026-04-06

**Authors:** Panche Taskov, Zorin Crainiceanu

**Affiliations:** University of Medicine and Pharmacy “Victor Babes”, Timisoara Romania / Plastic and Reconstructive Surgery Department Casa Austria, County Emergency Clinical Hospital “Pius Branzeu”, Timisoara Romania, Timisoara, Timis; University of Medicine and Pharmacy “Victor Babes”, Timisoara Romania / Plastic and Reconstructive Surgery Department Casa Austria, County Emergency Clinical Hospital “Pius Branzeu”, Timisoara Romania, Timisoara, Timis

## Abstract

**Introduction:**

Tangential excision and split-thickness grafting (SOC) remain the reference for deep burns but are blood-intensive, donor-site–limited and frequently yield hypertrophic scars. We evaluated a multimodal regenerative protocol (“PAN-EDNX”) coupling bromelain-based selective enzymatic debridement with autologous skin micro-grafts, platelet-rich plasma, microbial nanocellulose, cold plasma, 200 kDa hyaluronate dressings, AA based topics and structured psychological support.

**Methods:**

A single-centre retrospective study (2020-2025) compared consecutive adults with grade IIB/III burns treated 25.

by PAN-EDNX (n = 15) with size- and depth-matched SOC controls (n = 15). Primary end-points were time to complete eschar removal, need for secondary surgery, Vancouver Scar Scale (VSS) trajectory to 12 months, length of stay (LOS) and lost working days (LWD).

Continuous data were analysed with Welch’s t-test; categorical variables with Fisher’s exact test; correlations with Pearson’s r (α = 0.05).

**Results:**

Baseline age (35.3 ± 11.2 y vs 43.1 ± 7.8 y; *p*=.036) and %TBSA (36.3 ± 4.3 vs 33.2 ± 4.3; *p*=.056) were comparable; ABSI scores did not differ (*p*=.28).

PAN-EDNX halved eschar-clearance time (5.0 ± 1.1 vs 9.1 ± 1.5 d; *p*<.001) and eliminated secondary grafting (0 % vs 93 %; *p*<.001). Spontaneous epithelialisation occurred in every PAN-EDNX case vs 7 % of SOC (*p*<.001).

Mean LOS fell by 7.2 days (*p*=.006) and direct treatment costs by 22 % (*p*<.001).

One-year VSS was 1.6 ± 0.5 versus 4.9 ± 1.1 (*p*<.001). LWD were reduced by 45 % (60 ± 14 vs 110 ± 25 d; *p*=.002).

No procedure-related bleeding, electrolyte disorders or mortality differences were observed.

**Conclusions:**

The PAN-EDNX concept accelerates eschar clearance, obviates grafting, improves scar quality and shortens rehabilitation without compromising safety. Dermis preservation and micro-graft bio-stimulation appear synergistic, supporting wider integration of regenerative adjuncts into enzymatic debridement pathways for extensive burn.

**Applicability of Research to Practice:**

The clinical results obtained with therapeutic flowchart based on “PAN EDNX” demonstrate excellent aesthetic outcomes as well as functionality restoration.

This novel regenerative technique has shown promising results in burn healing process, reduced costs and hospitalization, decreased lost working days and increased quality of life.

“PAN EDNX” is a feasible, safe procedure and real CHALLENGE of personalized care in burn treatment.

**Funding for the study:**

N/A.

